# Pulmonary tumor embolism: A retrospective study over a 30-year period

**DOI:** 10.1371/journal.pone.0255917

**Published:** 2021-08-11

**Authors:** Xin He, Douglas C. Anthony, Zulmira Catoni, Weibiao Cao

**Affiliations:** 1 Department of Pathology and Laboratory Medicine, Rhode Island Hospital and The Alpert Medical School of Brown University, Providence, RI, United States of America; 2 Department of Pathology, University of Massachusetts School of Medicine, Worcester, MA, United States of America; 3 Department of Neurology, Rhode Island Hospital and The Alpert Medical School of Brown University, Providence, RI, United States of America; 4 Human Information Management, Rhode Island Hospital and The Alpert Medical School of Brown University, Providence, RI, United States of America; 5 Department of Medicine, Rhode Island Hospital and The Alpert Medical School of Brown University, Providence, RI, United States of America; University of Texas Medical Branch, UNITED STATES

## Abstract

**Background:**

Pulmonary tumor embolism (PTE) is difficult to detect before death, and it is unclear whether the discrepancy between antemortem clinical and postmortem diagnosis improves with the advance of the diagnostic technologies. In this study we determined the incidence of PTE and analyzed the discrepancy between antemortem clinical and postmortem diagnosis.

**Methods:**

We performed a retrospective autopsy study on patients with the history of malignant solid tumors from 1990 to 2020 and reviewed all the slides of the patients with PTE. We also analyzed the discrepancies between antemortem clinical and postmortem diagnosis in 1999, 2009 and 2019 by using the Goldman criteria. Goldman category major 1 refers to cases in which an autopsy diagnosis was the direct cause of death and was not recognized clinically, but if it had been recognized, it may have changed treatment or prolonged survival.

**Results:**

We found 20 (3%) cases with PTE out of the 658 autopsy cases with solid malignancies. Out of these 20 cases, urothelial carcinoma (30%, 6/20) and invasive ductal carcinoma of the breast (4/20, 20%) were the most common primary malignancies. Seven patients with shortness of breath died within 3–17 days (average 8.4±2.2 days) after onset of the symptoms. Pulmonary embolism was clinically suspected in seven out of twenty (35%, 7/20) patients before death, but only two patients (10, 2/20) were diagnosed by imaging studies before death. The rate of Goldman category major 1 was 13.2% (10/76) in 1999, 7.3% (4/55) in 2009 and 6.9% (8/116) in 2019. Although the rate of Goldman category major 1 appeared decreasing, the difference was not statistically significant. The autopsy rate was significantly higher in 2019 (8.4%, 116/1386) than in 2009 (4.4%, 55/1240).

**Conclusions:**

The incidence of PTE is uncommon. Despite the advances of the radiological techniques, radiological imaging studies did not detect the majority of PTEs. The discrepancy between the antemortem clinical and the postmortem diagnosis has not improved significantly over the past 30 years, emphasizing the value of autopsy.

## Introduction

Pulmonary tumor embolism is characterized by emboli in the pulmonary arterial system (including alveolar septal capillaries) consisting of tumor cells without fibrocellular intimal proliferation and not contiguous with the metastatic foci [1]. It is a rare event in the late stages of malignancy that carries a poor prognosis. Occlusion of the pulmonary microvasculature by tumor cells and associated thrombi may mimic thromboembolic disease. Detection of tumor cells in pulmonary vessels within the lung specimens is the only means to establish diagnosis. Unfortunately, microscopic pulmonary tumor embolism is rarely recognized before death [2] since 1) the clinical features of PTE are quite similar to those of thrombotic pulmonary embolism; 2) the difference between thrombotic and metastatic pulmonary embolism is not easily differentiated on radiographic studies, making radiological diagnosis difficult; and 3) PET/CT is not sensitive enough to differentiate tumor embolism from thrombotic embolism [3]. Therefore, pulmonary tumor embolism cases were identified primarily by autopsy.

Dr. Goldman evaluated the discrepancy between the antemortem clinical and autopsy diagnosis in 1983 [4] and proposed five categories. Dr. O’Connor and colleagues modified the classification in 2002 [5]. Category Major 1 refers to cases in which an autopsy diagnosis was the direct cause of death and was not recognized clinically, but if it had been recognized, it may have changed treatment or prolonged survival. The rate of category major 1 was 10% in Dr. Goldman’s study in 1983. It is not clear whether the discrepancy between the antemortem clinical and autopsy diagnosis improves over the past 30 years.

In this study, we performed a retrospective study to determine the incidence and origins of metastatic solid tumors leading to pulmonary tumor embolism over a 30-year period. We reported 20 cases with pulmonary tumor embolism and found that the incidence of pulmonary tumor embolism was 3% in our institution. We also analyzed the discrepancies between the antemortem clinical and the autopsy diagnosis in 1999, 2009 and 2019. The rate of Goldman category major 1 ranged from 6.9% to 13.2%.

## Methods

### Study design for pulmonary tumor embolism

The institutional review board (IRB) at Rhode Island Hospital approved this study (IRB committee number 1188347). No additional informed consent was required by the IRB for this chart review retrospective study.

**Fig 1:**
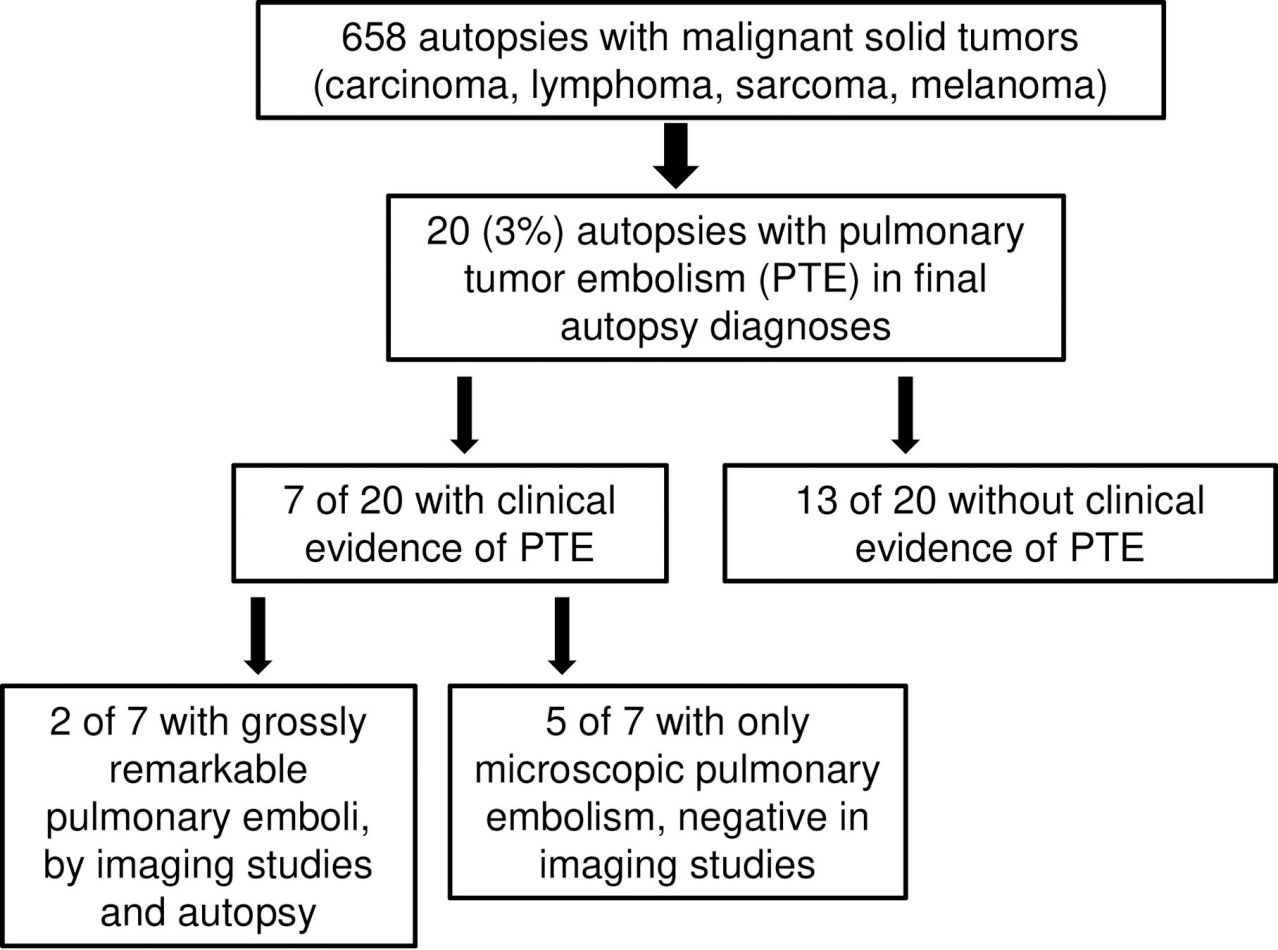
Flowchart of 658 autopsies with malignant solid tumors in our institution.

This retrospective study consisted of a chart review of autopsies at Rhode Island Hospital from 1990 to 2020. We reviewed autopsy cases with the history of malignant solid tumors (sarcomas, carcinomas, melanomas and lymphomas) and identified cases with pulmonary tumor embolism. The slides of lungs were re-reviewed to confirm the diagnosis of pulmonary tumor embolism. Pertinent clinical and pathologic data for pulmonary tumor embolism cases were collected (Fig 1).

Pulmonary tumor thrombotic microangiopathy is excluded from this study. Pulmonary tumor thrombotic microangiopathy is a related but different entity from pulmonary tumor embolism and is characterized by microscopic tumor cell emboli in the pulmonary microvasculature causing intimal proliferation and a syndrome of hypoxemia, pulmonary hypertension, right heart failure and death [6].

### Study design for discrepancies between antemortem clinical and autopsy diagnosis

In order to evaluate the agreement between antemortem clinical and autopsy diagnosis and to analyze how autopsy rate changed over the past 30 years, we reviewed the autopsy reports and the clinical histories of autopsy cases performed at Rhode Island Hospital in 1999, 2009 and 2019. Brain donation (most often for neurodegenerative disease) and medical examiner office cases were excluded. Discrepancies between antemortem clinical and autopsy diagnoses were classified using the modified Goldman criteria [5]. The total, inpatient and emergency department autopsy rates were also calculated.

### Statistical analysis

A Chi-square test was utilized to compare groups where appropriate.

## Results

### Pulmonary tumor embolism in autopsy cases with the history of malignant solid tumors in our institution

We identified 658 autopsy cases with postmortem diagnoses of malignant solid tumors in a period of 30 years from 1990 to 2020. Of the 658 autopsy cases, we found that 20 (3%) cases had pulmonary tumor emboli, including 11 males and 9 females. The average age at death for these 20 cases was 60.5±13 years (range 35–85 years). All cases were stage IV diseases with multiple distant organ metastases, including liver (70%, 14/20), lungs (70%, 14/20), bone (35%, 7/20), gastrointestinal tracts (25%, 5/20), omentum (20%, 4/20), adrenal glands (15%, 3/20), or others. Only one patient was on palliative chemotherapy before death. Two out of the twenty patients had a past medical history of thrombotic events. One patient had lower extremity thrombosis and the other had both lower extremity thrombosis and pulmonary embolism.

Of the 20 cases, urothelial carcinoma (n = 6, 30%) and invasive ductal carcinoma of the breast (n = 4, 20%) were the most common primary malignancies overall (Table 1), whereas in male patients urothelial carcinoma and gastric adenocarcinoma were the most common primaries and in female patients invasive ductal carcinoma of the breast and urothelial carcinoma of the bladder are the most common (Table 1). In addition, we analyzed the difference of primary tumors between the first 15 years (before 2005) and the second 15 years (after 2005). Urothelial carcinoma and colorectal adenocarcinoma were the most common primaries before 2005, whereas invasive ductal carcinoma of the breast and urothelial carcinoma of the bladder were the most common primaries in 2005 and after (Table 2).

**Table 1:** Primary malignancies in autopsy cases with pulmonary tumor embolism.

Primary Malignancies	N = 20 (%)	Male/Female	Age	Detected by imaging	Clinically suspected
Urothelial carcinoma of the bladder	6 (30)	3/3	61–85	0	2
Invasive ductal carcinoma of the breast	4 (20)	0/4	45–55	1	2
Colorectal adenocarcinoma	2 (10)	1/1	51–58	0	0
Gastric adenocarcinoma	2 (10)	2/0	40–62	0	0
Lung adenocarcinoma	1 (5)	1/0	48	0	0
Epithelioid angiosarcoma	1 (5)	1/0	73	0	1
Pancreatic adenocarcinoma	1 (5)	0/1	78	0	0
Hepatocellular carcinoma	1 (5)	1/0	66	0	0
Appendiceal adenocarcinoma	1 (5)	1/0	35	1	1
Prostatic adenocarcinoma	1 (5)	1/0	59	0	0

**Table 2:** Comparison of primary malignancies between before and after 2005.

Primary Malignancies	Before 2005 (N = 8, M/F = 5/3)	In and after 2005 (N = 12, M/F = 6/6)
Invasive ductal carcinoma of the breast	1	3
Urothelial carcinoma	2	4
Colorectal adenocarcinoma	2	0
Gastric adenocarcinoma	1	1
Pancreatic adenocarcinoma	0	1
Appendiceal adenocarcinoma	0	1
Angiosarcoma	0	1
Lung adenocarcinoma	1	0
Hepatocellular carcinoma	1	0
Prostatic adenocarcinoma	0	1

**Fig 2:**
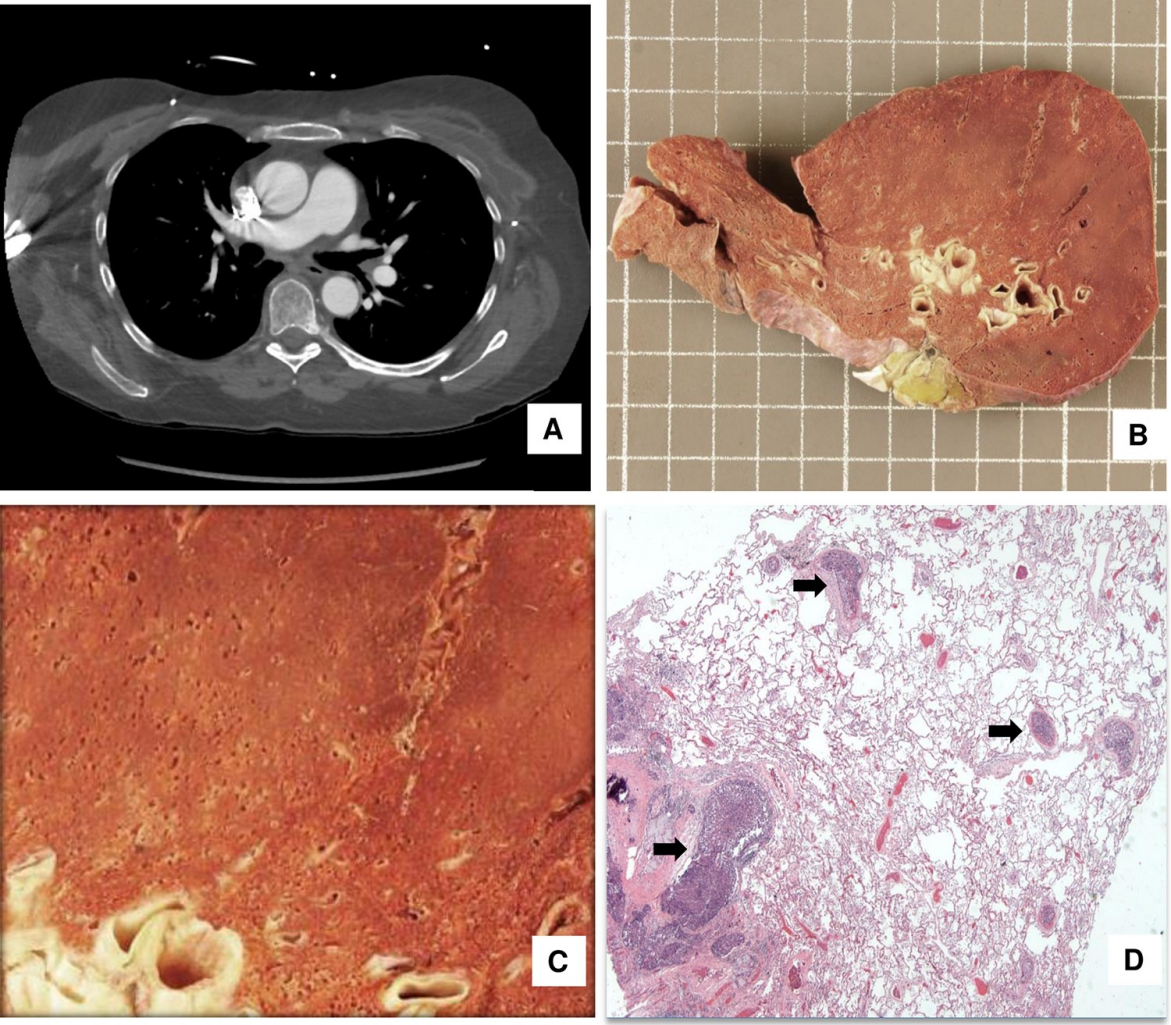
Representative case with microscopically but not grossly or radiologically detectable pulmonary tumor embolism in a patient with breast cancer. A. Radiographic image; B & C. Gross image of the lung; D. H&E image of the lung shows tumor emboli (arrows).

In 7 out of 20 (35%) patients with symptoms such as shortness of breath, hypoxemia and chest pain and clinically suspected of having pulmonary embolism before death, only one (5%) case was confirmed by imaging. In this case, postmortem gross examination demonstrated pulmonary emboli. Another patient without known symptoms had macroscopic tumor emboli detected by imaging studies and died suddenly. Therefore, the detection rate of pulmonary tumor embolism by the imaging studies was 10% (2/20) in our cohort. Eighteen out of twenty cases (90%, 18/20) only had microscopic pulmonary tumor emboli. For example, a 53-year-old female with history of breast cancer had microscopic pulmonary tumor emboli, which were not detected grossly or by imaging studies (Fig 2).

A majority (60%, 12 of 20) of the patients with pulmonary tumor embolism had no shortness of breath and/or hypoxemia. Among these patients, only microscopic but not macroscopic tumor emboli were found on autopsy. In 7 patients (35%, 7 of 20) with symptoms of shortness of breath and/or hypoxemia, 6 of them (85.7%, 6/7) had only microscopic pulmonary tumor emboli. These 7 patients died within 3–17 days (average 8.4±2.2 days) after the onset of shortness of breath.

These data indicate that the majority of pulmonary tumor emboli are not detectable by imaging and those with shortness of breath and/or hypoxemia died within days after symptom onset.

### Discrepancies between antemortem clinical and autopsy diagnoses in our institution

With the advances of technology in imaging, we studied whether the discrepancy between antemortem clinical and autopsy diagnosis improved over time. The percentage of Goldman category major 1 was 13.2% (10/76) in 1999, 7.3% (4/55) in 2009 and 6.9% (8/116) in 2019 (Table 3). Although the percentage appeared to be decreasing, the difference was not statistically significant.

**Table 3:** Goldman agreement classification between antemortem clinical diagnosis and autopsy diagnosis in 1999, 2009 and 2019.

	Major 1 Directly related to death; if recognized, may have altered treatment or survival	Major 2 Directly related to death; if recognized, would not have altered treatment or survival	Minor 3 Incidental autopsy finding not directly related to death but related to terminal disease process	Minor 4(i) Incidental autopsy finding unrelated to cause of death	Minor 4(ii) Incidental autopsy finding contributing to death in an already terminally ill patient	No discrepancy 5 Clinical and autopsy diagnoses in complete agreement
1999 (N = 76)	10 (13.2%, NS)	8 (10.5%)	0 (0%)	4 (5.3%)	2 (2.6%)	52 (68.4%)
2009 (N = 55)	4 (7.3%, NS)	5 (9.1%)	1 (1.8%)	6 (10.9%)	3 (5.5%)	36 (65.5%)
2019 (N = 116)	8 (6.9%, NS)	18 (15.5%)	0 (0%)	18 (15.5%)	6 (5.2%)	66 (56.9%)

Note: NS: No statistically significant difference between years

**Fig 3:**
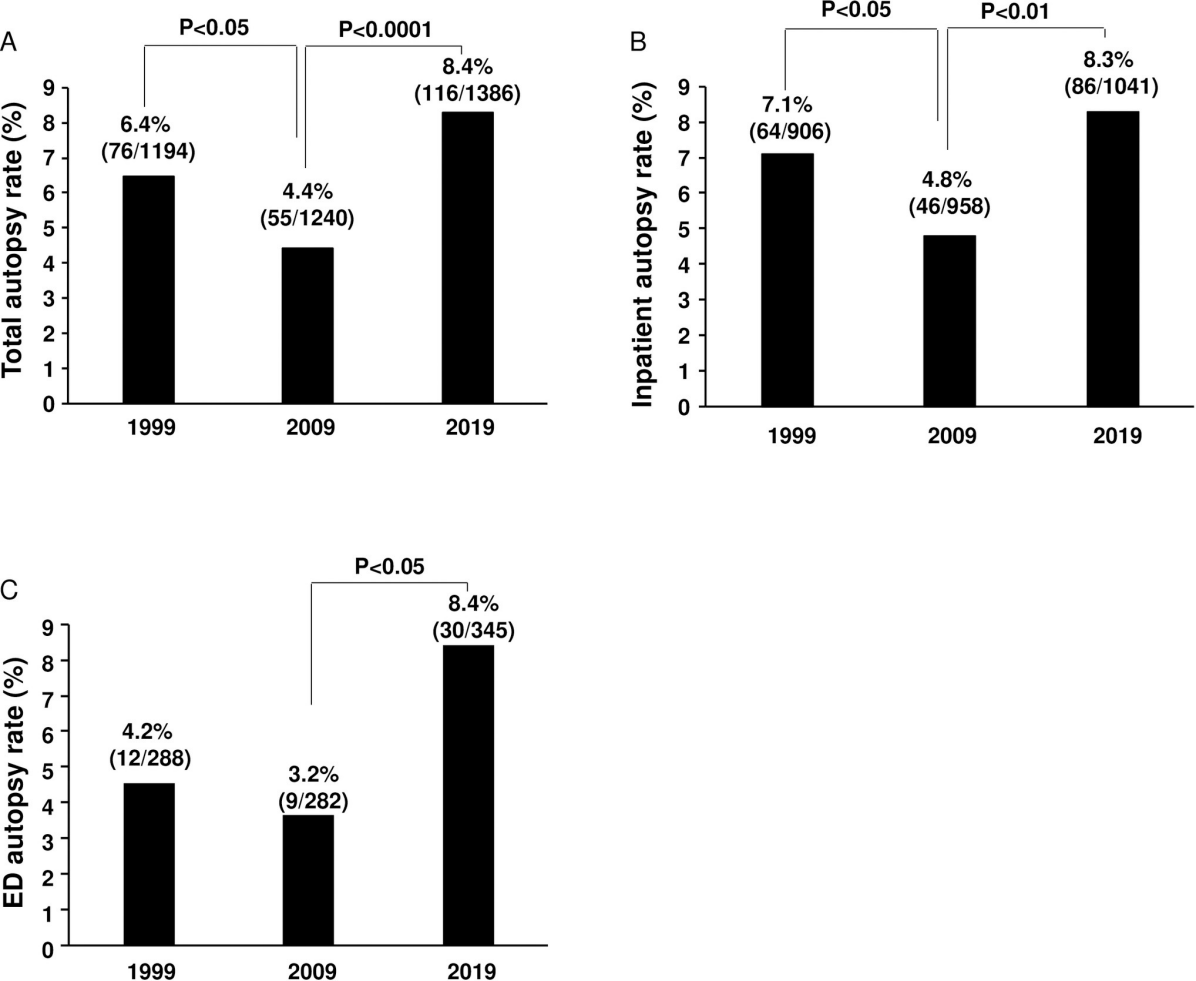
Autopsy rate in 1999, 2009 and 2019. A. The autopsy rate was significantly decreased in 2009 when compared with 1999 and significantly increased in 2019 when compared with 2009, but there was no significant difference between 1999 and 2019. B. The inpatient autopsy rate was significantly decreased in 2009 when compared with 1999 and significantly increased in 2019 when compared with 2009, but there was no significant difference between 1999 and 2019. C. The ED autopsy rate was significantly increased in 2019 when compared with 2009, but there was no significant difference between 1999 and 2019. These data demonstrate that the autopsy rate increase in 2019 was due to an increase of autopsy rate in both inpatients and ED patients.

We also analyzed the autopsy rate in 1999, 2009 and 2019. We found that the autopsy rate was 6.4% (76/1194) in 1999, 4.4% (55/1240) in 2009 and 8.4% (116/1386) in 2019. The autopsy rate was significantly decreased in 2009 compared with 1999 and significantly increased in 2019 when compared with 2009, but there was no significant difference between 1999 and 2019 (Fig 3A). We also analyzed the inpatient autopsy rate and the emergency department (ED) autopsy rate to determine whether either site was contributing more to the changes in autopsy rate over time. The inpatient autopsy rate was 7.1% (64/906) in 1999, 4.8% (46/958) in 2009 and 8.3% (86/1041) in 2019. Similar to the overall autopsy rate, the inpatient autopsy rate was significantly decreased in 2009 when compared with 1999 and significantly increased in 2019 when compared with 2009, but there was no significant difference between 1999 and 2019 (Fig 3B). The ED autopsy rate was 4.2% (12/288) in 1999, 3.2% (9/282) in 2009, and 8.4% (30/345) in 2019. The ED autopsy rate was significantly increased in 2019 when compared with 2009, but there was no significant difference between 1999 and 2019 (Fig 3C). These data suggest that the autopsy rate increase in 2019 was due to an increase in both the autopsy rate of the inpatients and that of the ED patients.

## Discussion

The incidence of pulmonary tumor embolism was 3% in our institution, which was slightly lower than that seen in literature which ranged from 3 to 26% of the autopsies in patients with solid tumors [2, 3, 7, 8]. The differences in the incidence could be due to the sampling, as most pulmonary tumor emboli are microscopic, and the more sections taken from the lungs, the higher the incidence of emboli are likely to be found. This study was a retrospective study, and we routinely take one section per lung lobe (at least 5 sections per case). Some prior papers, especially with prospective sampling, may perform greater sampling of the lungs.

Pulmonary tumor embolism may present with a wide range of symptoms, including acute hypoxia, chest and abdominal pain, cough, and indolent pulmonary hypertension [2]. In our institution, 60% (12/20) of the patients with pulmonary tumor embolism had no shortness of breath and/or hypoxemia; 35% (7/20) of patients had symptoms of shortness of breath and/or hypoxemia and died within 3–17 days (average 8.4±2.2 days) after the onset of shortness of breath.

Urothelial carcinoma was one of the most common primary malignancies in both male and female patients. Perhaps this is related to the fact that Rhode Island has the highest incidence of urothelial carcinoma in the United States [9].

**Fig 4:**
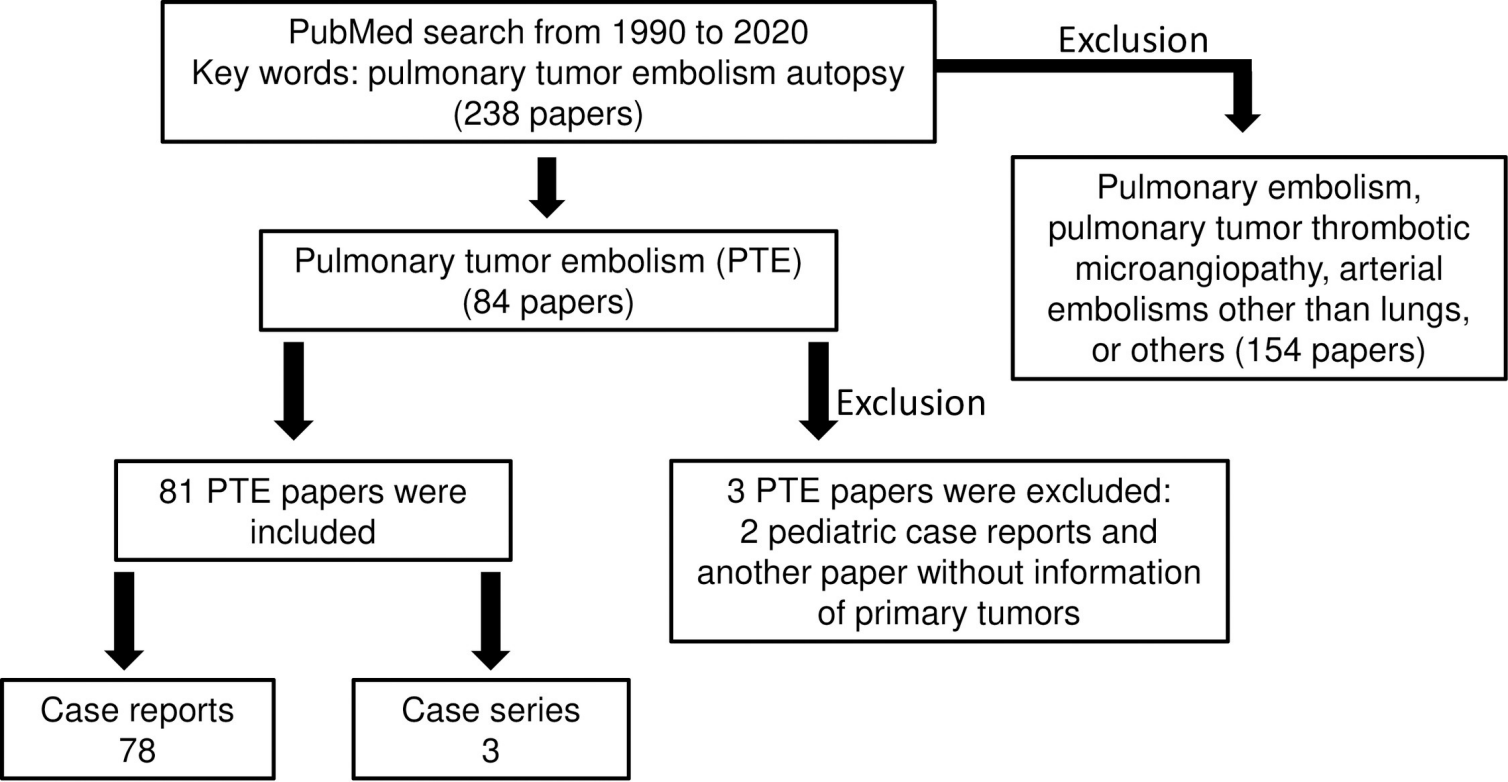
Flowchart of the literature review from 1990 to 2020.

To further gather the information about pulmonary tumor embolism, we performed an extensive literature review. Since our study focused on pulmonary tumor embolism in the autopsy cases, we searched PubMed from 1990 to 2020 by using key words “pulmonary tumor embolism and autopsy” and found 238 papers. Among these papers, 84 papers were related to pulmonary tumor embolism (Fig 4). Three papers were excluded, including two pediatric case reports [10, 11] as our study focused on adults, and one paper without primary tumor information [12]. The same authors of the latter [12] published two additional papers which were included in this study. Within 81 papers included, 78 were case reports and 3 were case series (7 to 19 cases).

In total, 120 cases with pulmonary tumor embolism were reported in the literature from 1990 to 2020 (Table 4). The most common primaries were breast cancer (14.2%, 17/120), gastric cancer (13.3%, 16/120), hepatocellular carcinoma (12.5%, 15/120), esophageal squamous cell carcinoma (7.5%, 9/120), lung cancer (5.8%, 7/120), urothelial carcinoma (5.8%, 7/120) and pancreatic adenocarcinoma (5%, 6/120). Before 2005, the most common primaries were hepatocellular carcinoma, gastric cancer and esophageal squamous cell carcinoma. After 2005, the most common primaries were breast cancer and gastric cancer. There was a regional difference because the majority of gastric cancer cases were from Japan.

**Table 4:** Pulmonary tumor embolism cases between 1990 and 2020 in the literature.

Primary Tumors	Sex (M/F/	Age (range/mean)	Total	Before 2005	After 2005	References
	Unknown)		N = 120			
Breast cancer	0/17/0	29–69 (49.8±5.7)	17	6	11	Kiljunen M, et al [15]; Lammi M, et al [16]; Kridel R, et al [17]; Nakamura H, et al [18]; Deeren D, et al [19]; Vlenterie M, et al [20]; Gajdos C [21]; Uga S, et al [22]; Veinot JP, et al [23]; van de Ven PJ, et al [24]; Kawasaki H, et al [25]; Moores LK, et al [26]; Soares FA, et al [27]
Gastric cancer	6/5/5	42–79 (63.4±4.8)	16	8	8	Tamura A and Matsubara O [28]; Abe H, et al [29]; Yoshii Y, et al [30]; Goshima H, et al [31]; Iwakami S, et al [32]; Matsuda H, et al [33]; Lo Priore E and Fusi-Schmidhauser T [34]; Kuraki T, et al [35]; Kato H, et al [36]; Koma Y, et al [37]
Hepatocellular carcinoma	5/0/10	41–65 (53.8±4.5)	15	12	3	Tamura A and Matsubara O [28]; Jäkel J, et al [38]; Chan GS, et al [39]; Clark T, et al [40]; Papp E, et al [41]; Gutiérrez-Macías A, et al [42]; Soares FA, et al [27]
Esophageal squamous cell carcinoma	1/0/8	N/A	9	8	1	Soares FA, et al [43]; Sentani K [44]; Soares FA, et al [27]
Lung cancer	2/0/5	59–69	7	4	3	Liang, YH et al [45]; Yilmaz S, et al [46]; Veinot JP, et al [23]; Soares FA, et al [27]; Nichols L, et al [47]
Urothelial carcinoma	7/0/0	66–86 (71.5±2.6)	7	5	2	de Escalante Yangüela B, et al [48]; Kitayama H, et al [49]; Scheppach W, et al [50]; Kobori G, et al [51]; Dhillon SS, et al [52]; Arisawa C, et al [53]; Suyama N, et al [54]
Pancreatic adenocarcinoma	1/0/5	41	6	6	0	Steiner S [55]; Soares FA, et al [27]; Bennink R, et al [56]; current 1 case
Unknown	0/2/3	30–56	5	4	1	Masoud SR, et al [57]; Kim AE, et al [58]; Weidemann DM, et al [59]; Soares FA, et al [27]
Colon cancer	1/2/1	57–76 (65±5.7)	4	1	3	Tamura A and Matsubara O [28]; Bergmann I, et al [60]; van der Burg-de Graauw NC and van Esser JW [61]
Uterine cervical cancer	0/4/0	57–66 (61±2.6)	4	1	3	Okazaki S, et al [62]; Tamura A and Matsubara O [28]; Vaideeswar P, et al [63]; Nakao Y, et al [64]
Prostate cancer	3/0/0	56–78 (70.7±7.3)	3	1	2	Lovrenski A et al [65]; Hattori T, et al [66]; Nakano M, et al [67]
Ovarian cancer	0/2/0	62	2	2	0	Veinot JP, et al [23]; Lambert-Jensen P, et al [68]
Gallbladder	1/1/0	63–67	2	2	0	Ando H, et al [69]; de Luis DA, et al [70]
Head neck squamous cell carcinoma	2/0/0	54–73	2	1	1	Uraguchi K, et al [71]; Kitaoka K, et al [72]
Female genital tract	0/2/0	N/A	2	2	0	Soares FA, et al [27]
Intrahepatic cholangiocarcinoma	0/1/0	70	1	0	1	Nakanishi D, et al [73]
Adenocarcinoma of small intestine	1/0/0	49	1	1	0	Nabeshima S, et al [74]
Testicular germ cell tumor	1/0/0	31	1	0	1	do Nascimento FB, et al [75]
Anaplastic thyroid cancer	0/1/0	88	1	1	0	Köppl H, et al [76]
Uterine cancer	0/1/0	70	1	0	1	Srettabunjong S and Chuangsuwanich T [77]
Mixed (RCC, gastric Cancer)	1/0/0	67	1	0	1	Fujiwara R, et al [78]
Chondrosarcoma	1/0/0	37	1	1	0	Mangiapan G, et al [79]
Thymic carcinoma	1/0/0	55	1	1	0	Sperling BL, et al [80]
Choriocarcinoma of the uterus	0/1/0	23	1	1	0	Chai L, et al [81]
Thyroid pleomorphic myxoid sarcoma	0/1/0	45	1	1	0	Grass H, et al [82]
Synovial sarcoma	1/0/0	52	1	0	1	Schmid S, et al [83]
Pelvic cancer	0/0/1	N/A	1	1	0	Tamura A and Matsubara O [28]
Extramammary Paget’s disease of left axilla	1/0/0	72	1	0	1	Oyama Y, et al [84]
Lymphoma	1/0/0	54	1	1	0	Skalidis EI, et al [85]
Testicular tumor	1/0/0	40	1	0	1	Hoshino A, et al [86]
Bone tumor	0/0/1	N/A	1	1	0	Soares FA, et al [27]
Angiosarcoma	0/1/0	76	1	0	1	Saitoh J, et al [87]
Malignant fibrous histiocytoma of the liver	1/0/0	43	1	1	0	Schweyer S, et al [88]
Renal cell carcinoma	0/0/1	N/A	1	1	0	Katz ES, et al [89]

Out of 81 papers, 51 case reports included the results of pre-mortem imaging studies. In 16 of them (31.4%, 16/51) the imaging studies detected pulmonary emboli. The detection rate by the imaging studies was 25.9% (7/27) before 2005, whereas the rate was 37.5% (9/24) after 2005. The difference was not statistically significant.

**Fig 5:**
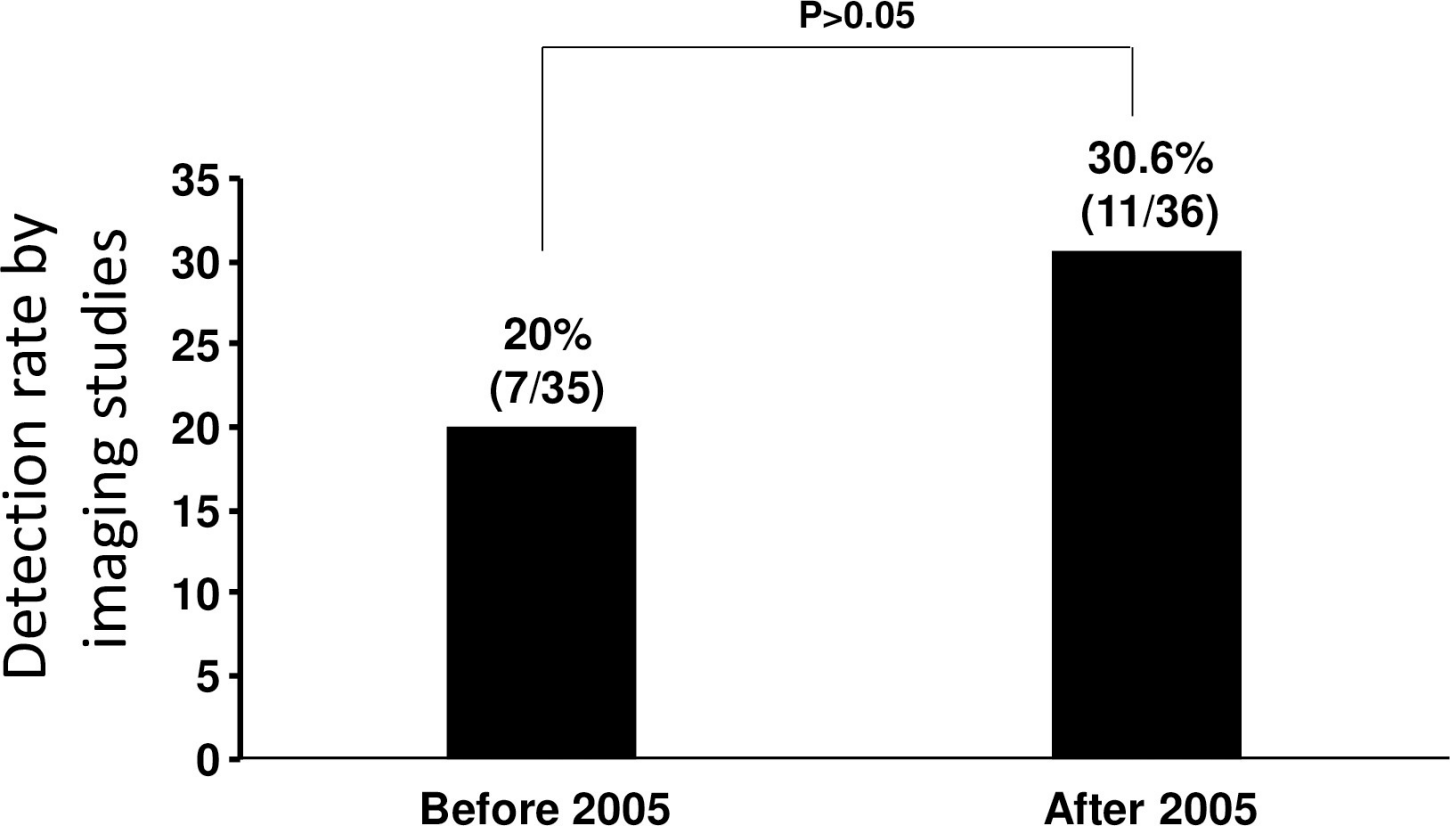
Detection rate of pulmonary tumor embolism. After incorporation of our own cases with the literature, the detection rate of pulmonary tumor emboli by the imaging studies was 20% (7/35) before 2005, whereas the rate was 30.6% (11/36) in and after 2005. The difference was not statistically significant (P>0.05).

After combining our cases with those in the literature, the most common primaries were breast cancer (15%, 21/140), gastric cancer (12.8%, 18/140), hepatocellular carcinoma (11.4%, 16/140), urothelial carcinoma (9.3%, 13/140), esophageal squamous cell carcinoma (6.4%, 9/140), lung cancer (5.7%, 8/140) and pancreatic adenocarcinoma (5%, 7/140). Before 2005, the most common primaries were hepatocellular carcinoma, gastric cancer, esophageal squamous cell carcinoma and breast cancer. After 2005, the most common primaries were breast cancer, gastric cancer and urothelial carcinoma (Table 5). The detection rate of pulmonary tumor embolism by imaging was 20% (7/35) before 2005 and 30.6% (11/36) in and after 2005. The difference was not statistically significant (Fig 5). Therefore, autopsy remains the “gold standard” for the diagnosis of pulmonary tumor embolism.

**Table 5:** Combination of pulmonary tumor embolism cases of our institution with the literature.

Primary Tumors	Sex (M/F/	Age (range/mean)	Total	Before 2005	After 2005	References
	Unknown)					
Breast cancer	0/21/0	29–69 (49.8±5.7)	21	7	14	Kiljunen M, et al [15]; Lammi M, et al [16]; Kridel R, et al [17]; Nakamura H, et al [18]; Deeren D, et al [19]; Vlenterie M, et al [20]; Gajdos C [21]; Uga S, et al [22]; Veinot JP, et al [23]; van de Ven PJ, et al [24]; Kawasaki H, et al [25]; Moores LK, et al [26]; Soares FA, et al [27]; current 4 cases
Gastric cancer	8/5/5	40–79 (61.3±4.4)	18	9	9	Tamura A and Matsubara O [28]; Abe H, et al [29]; Yoshii Y, et al [30]; Goshima H, et al [31]; Iwakami S, et al [32]; Matsuda H, et al [33]; Lo Priore E and Fusi-Schmidhauser T [34]; Kuraki T, et al [35]; Kato H, et al [36]; Koma Y, et al [37]; current 2 cases
Hepatocellular carcinoma	6/0/10	41–66 (52.8±4.2)	16	13	3	Tamura A and Matsubara O [28]; Jäkel J, et al [38]; Chan GS, et al [39]; Clark T, et al [40]; Papp E, et al [41]; Gutiérrez-Macías A, et al [42]; Soares FA, et al [27]; current 1 case
Urothelial carcinoma	10/3/0	61–86 (71.9±2)	13	7	6	de Escalante Yangüela B, et al [48]; Kitayama H, et al [49]; Scheppach W, et al [50]; Kobori G, et al [51]; Dhillon SS, et al [52]; Arisawa C, et al [53]; Suyama N, et al [54]; current 6 cases
Esophageal squamous cell carcinoma	1/0/8	N/A	9	8	1	Soares FA, et al [43]; Sentani K [44]; Soares FA, et al [27]
Lung cancer	3/0/5	48–69	8	2	1	Liang, YH et al [45]; Yilmaz S, et al [46]; Veinot JP, et al [23]; Soares FA, et al [27]; Nichols L, et al [47]current 1 case
		(58.7±6.1)				
Pancreatic adenocarcinoma	1/1/5	41–78	7	6	1	Steiner S [55]; Soares FA, et al [27]; Bennink R, et al [56]; current 1 case
Colon cancer	2/3/1	51–76 (60.8±4.2)	6	3	3	Tamura A and Matsubara O [28]; Bergmann I, et al [60]; van der Burg-de Graauw NC and van Esser JW [61]; current 2 cases
Unknown	0/2/3	30–56	5	4	1	Masoud SR, et al [57]; Kim AE, et al [58]; Weidemann DM, et al [59]; Soares FA, et al [27]
Uterine cervical cancer	0/4/0	57–66 (61±2.6)	4	1	3	Okazaki S, et al [62]; Tamura A and Matsubara O [28]; Vaideeswar P, et al [63]; Nakao Y, et al [64]
Prostate cancer	4/0/0	56–78 (67.7±5.9)	4	1	3	Lovrenski A et al [65]; Hattori T, et al [66]; Nakano M, et al [67]; current 1 case
Others (< = 2 per tumor)			39	28	11	

We also analyzed the discrepancies between antemortem clinical and autopsy diagnosis in our cohort by using the modified Goldman criteria [5]. We found that the percentage of Goldman category major 1 showed a tendency toward decreasing, but the difference was not statistically significant. The incidence of category major 1 was 6.9–13.2% in our study over the time period, which is similar to 10% reported in Dr. Goldman’s study in 1983 [4] and 7% in Dr. O’Connor’s study in 2002 [5]. Our data suggest that the percentage of category major 1 of the discrepancies between antemortem clinical and autopsy diagnosis has not changed significantly over the past 30 years.

The autopsy rate in the US and other western nations has decreased. The autopsy rate in academic hospitals is approximately 10%, while many non-teaching hospitals no longer perform autopsies [13, 14]. The autopsy rate at Rhode Island Hospital in 2019 was significantly higher than 2009, likely due to an increase in the rates for both inpatients and ED patients. The increase of autopsy rate may be due to an implementation of EPIC electronic health record system in our hospital in 2015, which requires the clinical team completing the death certificate to answer whether autopsy has been offered.

In conclusion, the majority of pulmonary tumor emboli cannot be detected radiographically and the autopsy remains the “gold standard” for the diagnosis of pulmonary tumor embolism. Although technologies in radiology have improved remarkably over the past 30 years, the discrepancies between antemortem clinical and autopsy diagnosis do not differ significantly.

## Abbreviations

PTE: pulmonary tumor embolism
ED: Emergency Department
